# Isolation of Aquatic Plant Growth-Promoting Bacteria for the Floating Plant Duckweed (*Lemna minor*)

**DOI:** 10.3390/microorganisms10081564

**Published:** 2022-08-03

**Authors:** Ayaka Makino, Ryosuke Nakai, Yasuko Yoneda, Tadashi Toyama, Yasuhiro Tanaka, Xian-Ying Meng, Kazuhiro Mori, Michihiko Ike, Masaaki Morikawa, Yoichi Kamagata, Hideyuki Tamaki

**Affiliations:** 1 Bioproduction Research Institute, National Institute of Advanced Industrial Science and Technology (AIST), Sapporo 062-8517, Hokkaido, Japan; a-makino@aist.go.jp (A.M.); nakai-ryosuke@aist.go.jp (R.N.); 2 Bioproduction Research Institute, National Institute of Advanced Industrial Science and Technology (AIST), Tsukuba 305-8566, Ibaraki, Japan; yndyasko@gmail.com (Y.Y.); y-mou@aist.go.jp (X.-Y.M.); y.kamagata@aist.go.jp (Y.K.); 3 Graduate School of Engineering, University of Yamanashi, Kofu 400-8511, Yamanashi, Japan; ttohyama@yamanashi.ac.jp (T.T.); mori@yamanashi.ac.jp (K.M.); 4 Graduate School of Life and Environmental Sciences, University of Yamanashi, Kofu 400-8510, Yamanashi, Japan; yasuhiro@yamanashi.ac.jp; 5 Graduate School of Engineering, Osaka University, Suita 565-0871, Osaka, Japan; ike@see.eng.osaka-u.ac.jp; 6 Graduate School of Environmental Science, Hokkaido University, Sapporo 060-0810, Hokkaido, Japan; morikawa@ees.hokudai.ac.jp; 7 Faculty of Life and Environmental Sciences, University of Tsukuba, Tsukuba 305-8577, Ibaraki, Japan; 8 Biotechnology Research Center, University of Tokyo, Bunkyo-ku, Tokyo 113-0032, Japan

**Keywords:** isolation, cultivation, plant growth-promoting bacteria, *Pelomonas*, duckweeds

## Abstract

Plant growth-promoting bacteria (PGPB) can exert beneficial growth effects on their host plants. Little is known about the phylogeny and growth-promoting mechanisms of PGPB associated with aquatic plants, although those of terrestrial PGPB have been well-studied. Here, we report four novel aquatic PGPB strains, MRB1–4 (NITE P-01645–P-01648), for duckweed *Lemna minor* from our rhizobacterial collection isolated from *Lythrum anceps*. The number of *L. minor* fronds during 14 days co-culture with the strains MRB1–4 increased by 2.1–3.8-fold, compared with an uninoculated control; the plant biomass and chlorophyll content in co-cultures also increased. Moreover, all strains possessed an indole-3-acetic acid production trait in common with a plant growth-promoting trait of terrestrial PGPB. Phylogenetic analysis showed that three strains, MRB-1, -3, and -4, were affiliated with known proteobacterial genera (*Bradyrhizobium* and *Pelomonas*); this report is the first to describe a plant-growth promoting activity of *Pelomonas* members. The gammaproteobacterial strain MRB2 was suggested to be phylogenetically novel at the genus level. Under microscopic observation, the *Pelomonas* strain MRB3 was epiphytic and adhered to both the root surfaces and fronds of duckweed. The duckweed PGPB obtained here could serve as a new model for understanding unforeseen mechanisms behind aquatic plant-microbe interactions.

## 1. Introduction

All plants in nature interact with an astonishing variety of microorganisms, and plant-associated bacteria can impart deleterious, neutral, or beneficial effects on plant growth and yield [1,2]. A typical example of beneficial bacteria are the plant growth-promoting bacteria (PGPB) [3]. PGPB for terrestrial plants encompass diverse but specific bacterial groups such as *Azospirillum*, *Bacillus*, *Burkholderia*, *Bradyrhizobium*, *Pseudomonas*, and *Serratia*; these strains have been utilized to improve the growth of terrestrial agricultural crops in greenhouses and field trials [4,5,6]. The utilization of PGPB (also known as biofertilizers) has been suggested as an “eco-friendly” alternative to chemical fertilizers and pesticides.

Duckweeds are tiny floating plants that have attracted attention due to their starch-rich biomass, high-protein contents [7,8], and high yields of biofuels (e.g., bio-ethanol [9,10]). Importantly, the biomass yield of duckweeds is comparable to that of certain algae that are considered high-potential energy crops [11,12]. These attractive features have promoted efforts to improve the productivity and culture performance of duckweeds [13,14,15]. As part of this effort, the PGPB for duckweeds, *Acinetobacter calcoaceticus* P23, which increases the number of duckweed fronds (leaf-like structures), was isolated from *Lemna aoukikusa* (indistinguishable from *Lemna aequinoctialis* as reported by Borisjuk et al. [16]) [17]. *A. calcoaceticus* P23 also has the unique abilities to colonize mainly the duckweed fronds [18] and to enhance the host chlorophyll production [19].

Through cultivation efforts, several other PGPB for duckweeds have also been reported, including *Bacillus amyloliquefaciens* FZB42 [20], *Exiguobacterium* sp. MH3 [21], *Aquitalea magnusonii* H3 [22], *Pseudomonas* strains [18,22], *Ensifer* sp. SP4 [23] and the *Acidobacteria* strains recently isolated by our group [24]. In contrast, a culture-independent study showed that aquatic plants, including duckweed, harbor diverse uncharacterized bacterial taxa [25,26,27]. Crump and Koch [25] also observed unique bacterial members of the *Bacteroidetes*, *Proteobacteria*, and *Spirochaetes* phyla distributed broadly among aquatic angiosperms. These investigations indicate that additional candidate PGPB for duckweeds could be obtained by screening bacterial isolates from diverse aquatic plants.

There are a few reports on the mechanisms of the symbiotic effects of PGPB in duckweeds. One study suggested that indole-3-acetic acid (IAA) produced by *B. amyloliquefaciens* FZB42 is a major growth-promoting factor for *Lemna minor* [20], while another study demonstrated that exogeneous IAA had no apparent positive effect on duckweed growth [24,28]. These inconsistent results suggest that mechanisms other than IAA production are associated with enhancement of host growth. More importantly, it is unclear whether the plant growth-promoting (PGP) traits (i.e., siderophore productivity and phosphate-solubilizing capability; [29]) found in terrestrial PGPB are also observed in aquatic PGPB.

Here, we report four novel PGPB for duckweeds from our collection of rhizobacteria isolated from various aquatic plants. The bacterial isolates were tested for their growth-promoting effect by co-cultivation with aseptic duckweed (*L. minor*). The duckweed *L. minor* was used herein because it is widely used as a model aquatic plant in physiological and molecular analyses and it has been used in biotechnological applications [30]. After the screening, the symbiotic effects of the selected PGPB strains on the dry weight and chlorophyll concentration of *L. minor* were evaluated. In addition, other PGP traits of the strains, including the root colonization ability, were also assessed.

## 2. Materials and Methods

### 2.1. Isolation and Cultivation of Rhizobacteria from Aquatic Plants

The aquatic plants *Phragmites australis*, *Lythrum anceps*, *Zizania latifolia*, *Limnobium laevigatum*, *Salvinia molesta*, *Spirodela polyrhiza*, and *L. minor* were harvested from a pond located within the Yamanashi prefectural wood park, central Japan (35°38′23″ N, 138°40′36″ E) and then used as isolation sources of rhizobacteria. The limnological features of the pond are described in previous studies [27,31]. An approximately 0.15 g (wet weight) sample of the plant roots was rinsed twice with 30 mL of sterilized modified Hoagland nutrient solution [32] (hereinafter mHoagland solution) in a 50-mL test tube to remove the microorganisms loosely attached to the plants following a previous study [31]. Then, roots from each plant were mechanically homogenized in 10 mL of sterilized mHoagland solution with an Ace HOMOGENIZER AM-1 (Nihonseiki, Tokyo, Japan) as described in a previous study [27]. The homogenates were serially diluted (10-fold) with mHoagland solution (pH 7.0). A sample of each dilution (50 µL) was independently spread on 1/10 diluted R2A plates (hereinafter R2A-PS plates), which are generally used for the cultivation of heterotrophic bacteria in the environment [33]. The R2A-PS plates were prepared by autoclaving the phosphate and agar separately to mitigate oxidative stress [34]. The R2A-PS plates were solidified with two gelling agents, 1.5% agar and 1.0% gellan gum, for the isolation of a wider variety of microorganisms, as described by Tamaki et al. [35]. After incubation at 25 °C in the dark, single colonies were picked and streaked onto fresh plates to purify the colony-forming isolates. More than 100 isolates were obtained in this manner and preserved in 13% glycerol at −80 °C until further analysis.

### 2.2. Screening of Aquatic PGPB for Duckweeds

To screen PGPB for duckweeds, an experiment utilizing co-cultivation of duckweeds and bacteria was designed. An aseptic *L. minor* culture was prepared as described previously [36]. In brief, the duckweeds were routinely cultivated in 300-mL flasks containing 150 mL of sterilized mHoagland solution at 25 °C under a 16-h/8-h day/night photoperiod at 5000 lux. Approximately one-third of the duckweed individuals in each flask were transplanted into fresh mHoagland medium once per week to maintain fresh duckweeds. An aseptic duckweed was transferred to a flat test-tube (40φ × 130 mm) containing 40 mL of mHoagland medium. Rhizobacterial isolates were inoculated directly from glycerol stocks onto R2A-PS agar plates, as described above, at 25 °C in the dark. Bacterial colonies were swabbed from the R2A-PS plate after one week of incubation and then suspended in two fronds of *L. minor* culture to a final OD_600_ of 0.3. The duckweed/isolate co-cultures were incubated for 14 days under the same conditions as used for routine cultivation. A negative control with no bacterial inoculation and a positive control co-cultured with *A. calcoaceticus* P23, a well-known PGPB for duckweeds [17], were also tested for this experiment. After the cultivation, the number of *L. minor* fronds was counted to evaluate the growth-promoting activity, and the treated/control ratio was calculated based on the frond number of the negative control. In this study, the isolates showing growth-promoting activity at a treated/control ratio ≥ 2 were defined as PGPB for duckweeds. The PGPB strains that reproducibly exceeded this ratio were used for further evaluation.

### 2.3. Evaluation of Symbiotic Effects in the Duckweed/PGPB Co-Culture

To assess the symbiotic effects of PGPB strains on duckweeds, a co-culture experiment was performed in a larger flask than was used for the initial screening. An aseptic *L. minor* culture was transferred to a 300-mL flask containing 150 mL of mHoagland medium. Each PGPB strain was prepared in the same manner as for the initial screening, and then inoculated to 10 fronds of *L. minor* culture. The duckweed/PGPB co-cultures were incubated for 14 days in triplicate. A negative control with no bacterial inoculation and a positive control co-cultured with *A. calcoaceticus* P23 were tested. During the cultivation period, the number of *L. minor* fronds was counted on days 0, 3, 7, 10, and 14. The duckweed samples were collected after the 14-day cultivation period, and then dried at 70 °C for 24 h and weighed for biomass determination in triplicate. Chlorophyll in the dried samples was extracted with 5 mL of *N*,*N*-dimethylformamide at 4 °C in the dark for 24 h. After centrifugation at 15,000× *g* at 4 °C for 1 min, absorbances of the solvents were measured at wavelengths of 646 nm and 663 nm. The chlorophyll content was calculated using the following equation: Chl *a* + *b* = 17.67A_646.8_ + 7.12A_663.8_ [37], and was expressed as mg chlorophyll per 100 g of frond dry weight.

### 2.4. Phylogenetic Identification of PGPB Strains

Genomic DNA of the PGPB was extracted from colonies picked from R2A-PS agar plates using a Fast-DNA Spin Kit for Soil (MP-Biomedicals, Tokyo, Japan) according to the manufacturer’s instruction. PCR amplification of the 16S rRNA gene was performed with the universal bacterial primers 10F (5′-AGAGTTTGATCMTGGCTCAG-3′) and 1492R (5′-TACGGYTACCTTGTTACGACTT-3′) and *TaKaRa Ex Taq* DNA polymerase and accompanying reagents (TaKaRa, Otsu, Japan). Each PCR was carried out in a 50 µL reaction volume using a thermal cycler (TaKaRa PCR Thermal Cycler Dice Gradient TP600; TaKaRa) under the following cycling conditions: an initial denaturation step at 95 °C for 2 min, followed by 35 cycles of 95 °C for 30 s, 56 °C for 30 s, and 72 °C for 1.5 min. The expected size (approximately 1500 bp) of PCR products was checked by electrophoresis on a 1.5% agarose gel. The amplified products were purified using an Agencourt AMPure XP system (Beckman Coulter, Tokyo, Japan) on a Biomek 3000 workstation (Beckman Coulter) according to the manufacturer’s instructions. The purified products were cycle-sequenced with a Big Dye Terminator v3.1 Cycle Sequencing Kit (Applied Biosystems, Tokyo, Japan); the sequencing was performed with primers 10F (the same sequence described above), 787F (5′-ATTAGATACCCNGGTAG-3′), 909F (5′-ACTYAAAKGAATTGRCGGGG-3′), 907R (5′-CCGYCAATTCMTTTRAGTTT-3′), and 1492R (described above) under the following cycling conditions: an initial denaturation step at 96 °C for 1 min, followed by 25 cycles of denaturation at 96 °C for 10 s, annealing at 50° C for 5 s, and a final extension step at 60 °C for 4 min. The sequencing products were purified using an Agencourt CleanSEQ (Beckman Coulter) on a Biomek 3000 workstation, and DNA sequencing was performed with an ABI 3130xl Genetic Analyzer (Applied Biosystems). The obtained 16S rRNA gene sequence (approximately 1300 bp) was compared with the EzBioCloud database (https://www.ezbiocloud.net/ [accessed on 2 May 2022]) [38] using a BLASTN search and pairwise sequence alignment [39]. The sequence data have been deposited in the DDBJ/ENA/NCBI databases under accession numbers LC710946 to LC710949. For potentially novel species/genera, the sequence was aligned with related sequences identified by an ExBioCloud database search using ClustalW [40]. A phylogenetic tree was then constructed using the neighbor-joining method [41] in MEGA X [42].

### 2.5. Assays on Plant Growth-Promoting Properties and Motility of PGPB Strains

Plant growth-promoting (PGP) traits (IAA production, phosphate solubilization, siderophore production and nitrogen fixation) of the four newly obtained PGPB strains were examined using conventional methods; these traits have been well studied for terrestrial PGPB [29] but not aquatic ones [24]. IAA production was determined using Salkowski’s method [43]. Briefly, each PGPB strain was inoculated into 5 mL of R2A liquid medium supplemented with l-tryptophan (as a precursor of IAA) in a 15-mL test tube; the inoculums were incubated at 28 °C with shaking at 125 rpm for 2 days in the dark. After cultivation, the liquid culture was centrifuged at 5000× *g* for 10 min at 24 °C. Then, 750 μL of Salkowski reagent (a mixture of 2 mL of 0.5 M FeCl_3_ and 98 mL of 35% perchloric acid) was added to 500 μL of culture supernatant, and the mixture was incubated for 10 min at 24 °C in the dark. IAA production activity was determined by the naked eye as a color change from pink to red. Phosphate solubilization activity was evaluated using Pikovskaya’s agar medium [44]. Each PGPB strain grown in R2A liquid medium was spot inoculated onto the agar plates and incubated at 25 °C for 7 days. The solubilization activity was observed as a clear zone (halo) around the colonies. Siderophore production activity was assessed using chrome azurol S (CAS) agar medium [45]. Each PGPB strain grown in R2A liquid medium was spot inoculated onto the CAS plates and incubated at 25 °C for 7 days. The production activity was visualized as a change in the halo color from blue to yellow. Nitrogen fixation potential was tested by direct PCR, cloning, and sequencing of the nitrogenase gene (*nifH*). The PCR was conducted with the primer set IGK3 (5′-GCIWTHTAYGGIAARGGIGGIATHGGIAA-3′) and DVV (5′-CTRCAICAIACRCCICCIAARCGITA-3′) [46] using the reaction conditions reported in a previous study [47], with the exception of annealing temperature (annealing was performed at 58 °C herein). The PCR products were cloned into pCR4-TOPO vector (TOPO TA cloning kit for sequencing; ThermoFisher, Tokyo, Japan) and sequenced. The obtained sequences were compared with the NCBI nr/nt database using a BLAST search. Further, growth in Barraquio’s nitrogen-free basal medium [48] was tested under a microaerobic condition by using an Anaero Pack-Microaero system (note that this pack contains an oxygen-to-carbon dioxide-transformation reagent; Mitsubishi Gas Chemical, Tokyo, Japan) with a hydrogen-generating reagent (Mitsubishi Gas Chemical). The motility of each PGPB strain was assessed by direct observation using phase-contrast microscopy (Axio Observer Z1; ZEISS, Tokyo, Japan).

### 2.6. Microscopic Observations of PGPB Strains on Duckweed Surface

The duckweed samples co-cultured with each PGPB strain were picked at days 1, 3, 7, 10, and 14 and stained with 10 µL each of the SYTO 9 and propidium iodide solutions from a LIVE/DEAD BacLight Bacterial Viability Kit (ThermoFisher) on glass slides for 10 min in the dark. PGPB cells attached to duckweed roots were observed using a fluorescence microscope (Axio Observer Z1). The cells were distinguished by labeling in green using SYTO 9 for viable cells or in red using propidium iodide for dead cells. For the PGPB that showed strong adhesion to the duckweed root, the following electron microscopic observation was further performed. The duckweed sample co-cultured with PGPB was picked from the tube at days 3 and 7 and fixed with 2% (*v*/*v*) glutaraldehyde in 0.10 M phosphate buffer (pH 7.4) at 4 °C for 2 h. After rinsing three times with 0.10 M phosphate buffer, the sample was post-fixed with 1% osmium tetroxide at 4 °C for 90 min, and was then dehydrated with a graded series of ethanol (50%, 70%, 90%, 95%, and 100%) at room temperature. The sample was dried using a critical-point drying apparatus (JCPD-5; JEOL, Tokyo, Japan) and was then coated with osmium in an osmium plasma coater (Neoc-Pro; Meiwafosis, Tokyo, Japan). Finally, the sample was observed using a scanning electron microscope (FE-SEM, S4500; Hitachi, Tokyo, Japan).

### 2.7. Statistical Analysis

Each value used in the statistical analysis represents the results from triplicate experiments. All results are expressed as mean ± SD. Significance (*p* < 0.05) by *t*-test was calculated versus uninoculated control or individual inoculations.

## 3. Results and Discussion

### 3.1. Duckweed Growth Promotion by Aquatic PGPB

**Figure 1:**
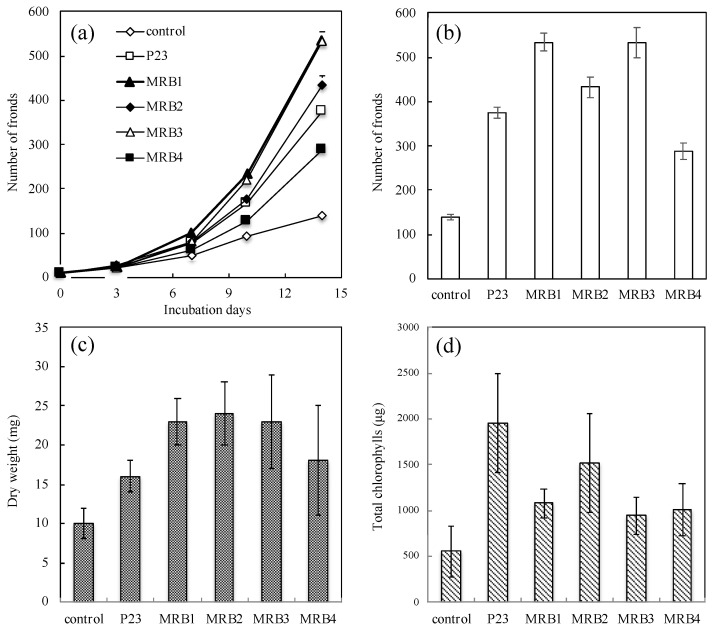
Symbiotic effects of the obtained strains MRB1–4 on the frond number, dry weight biomass, and chlorophyll content of duckweed (*Lemna minor*) after co-cultivation. The panels show changes in the (**a**) number of duckweed fronds over 14 days, (**b**) number and (**c**) dry weight biomass of duckweed fronds and (**d**) total chlorophyll content expressed as mg chlorophyll per 100 g frond dry weight after 14-day culture with no bacterial inoculation (labeled as control), *Acinetobacter calcoaceticus* P23, a known PGPB as a positive control strain (labeled as P23), and our strains MRB1–4. Each value represents the mean ± standard deviation (SD) of triplicate experiments.

More than 100 rhizospheric strains were isolated from seven aquatic plants (*P. australis*, *L. anceps*, *Z. latifolia*, *L. laevigatum*, *S. molesta*, *S. polyrrhiza* and *L. minor*). In the initial experiment in a test-tube, a growth-promoting effect on duckweed (*L. minor*) was evaluated as an increase in total frond number by co-cultivation with aseptic duckweed according to the previous study [17]. Four PGPB strains, which reproducibly increased the number of duckweed fronds by more than two-fold compared to that in the aseptic *L. minor*, were screened and selected for further studies. These aquatic PGPB strains were all isolated from *Japanese loosestrife* (*L. anceps*; Japanese name: “Miso-hagi”) and designated MRB (from Miso-hagi rhizobacteria) isolates 1 to 4. In the second experiment in a large flask, the frond numbers of *L. minor* co-cultivated with MRB1, MRB2, MRB3, and MRB4 were markedly increased from an initial number of 10 fronds to 534 ± 20 fronds (*p* < 0.05; all *p*-values by *t*-test were calculated versus the uninoculated control), 433 ± 23 fronds (*p* < 0.05), 531 ± 35 fronds (*p* < 0.01), and 289 ± 19 fronds (*p* < 0.05), respectively; in the uninoculated control, the numbers increased to only 140 ± 6 fronds (Figure 1a,b). These treated/control ratios (~3.8) of frond numbers were larger than the number (2.7) from *L. minor* co-cultured with *A. calcoaceticus* P23 (subsequently designated as “*Lemna*/P23”) as a positive control treatment (375 ± 13 fronds, *p* < 0.05), except in the case of the *Lemna*/MRB4 data. Note that the *Lemna*/MRB4 data were significantly different (*p* < 0.05) from the other *Lemna*/MRB1, *Lemna*/MRB2, and *Lemna*/MRB3 data. This result demonstrates that our PGPB strains promote host plant growth, and especially frond multiplication.

We further compared the biomass and chlorophyll content of *L. minor* in co-culture with and without each PGPB strain. The dry weights of *Lemna*/MRB1, *Lemna*/MRB2, *Lemna*/MRB3, and *Lemna*/MRB4 co-cultivations were 23 ± 3 mg (*p* < 0.05; all p-values by t-test were calculated versus the control), 24 ± 4 mg (*p* < 0.05), 23 ± 5 mg (*p* < 0.05), and 18 ± 7 mg (*p* > 0.1), respectively (Figure 1c). These values were 1.8- to 2.4-fold higher than that of the uninoculated control (10 ± 2 mg dry weight) and were also higher than that of *Lemna*/P23 (16 ± 2 mg dry weight, *p* < 0.05). In contrast, the effects on chlorophyll content varied among the strains examined. The total chlorophyll contents per 100 g of dry weight in the co-cultures with MRB1, MRB2, MRB3, and MRB4 were 1076 ± 163 μg (*p* = 0.05), 1516 ± 537 μg (*p* = 0.05), 947 ± 278 μg (*p* > 0.1), and 966 ± 278 μg (*p* > 0.1), respectively (Figure 1d). The treated/control ratio (2.7) of MRB2 was by far the highest among our PGPB strains, while the best enhancement was observed in *Lemna*/P23 (3.5). Note that no significant differences were found in the increase of biomass or chlorophyll content in the *Lemna*/MRB1 to *Lemna*/MRB4 data. Chlorophyll content enhancements by aquatic PGPB have also been reported in our recent studies [19,23,24]. Similar enhancement effects by PGPB have also been observed in terrestrial plants such as green gram [49,50], maize [51,52], tomato [53,54], peanut [55], and rice [56]. Changes in chlorophyll content may affect the host photosynthetic activity and its associated growth rate, but the frond number and its dry weight in *Lemna*/P23 were not particularly elevated (Figure 1a–c). Thus, no clear correlation between increased chlorophyll and biomass was observed; the PGPB studied here might have different PGP effects on *L. minor*.

**Figure 2:**
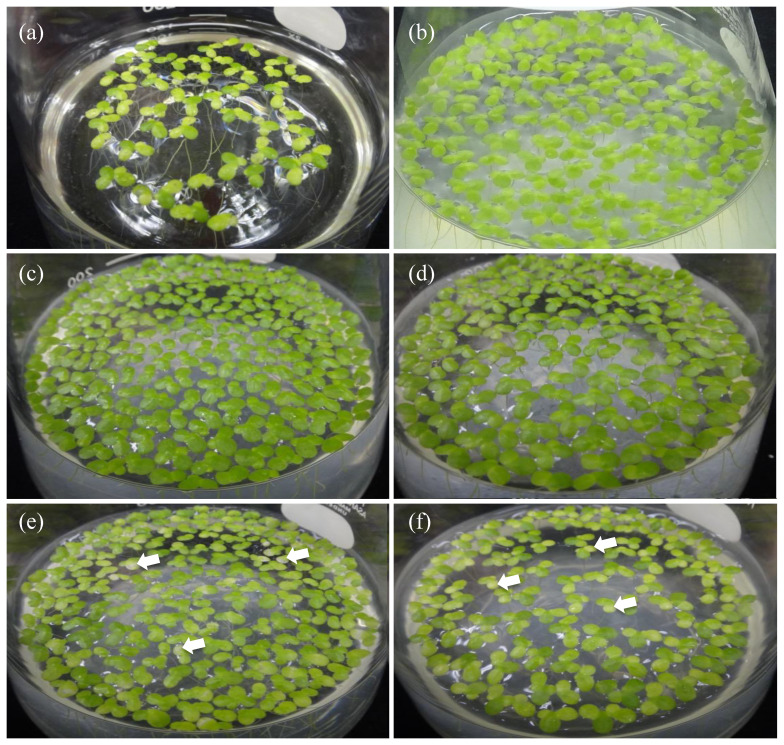
Growth-promotion effects of the obtained strains MRB1–4 on duckweed fronds after co-cultivation. The photographs show (**a**) aseptic duckweed, and aseptic duckweed co-cultured on day 14 with (**b**) *Acinetobacter calcoaceticus* P23, a known PGPB, as a positive control strain, or with our strains (**c**) MRB1, (**d**) MRB2, (**e**) MRB3, and (**f**) MRB4. The typical light-colored daughter fronds are indicated by white arrows.

To be consistent with the chlorophyll content enhancement, we note that the intensity of the green color of the *L. minor* fronds co-cultured with *A. calcoaceticus* P23, as well as that of our strains MRB1 and MRB2, was much greater than that of the aseptic control (Figure 2). By contrast, other strains, MRB3 and MRB4, showed no significant difference relative to the aseptic control, and the green color of their daughter fronds was lighter than that of the mother fronds (Figure 2e,f). The decrease in chlorophyll of such daughter fronds might have affected the overall chlorophyll content.

### 3.2. Phylogenetic Identification of Aquatic PGPB

Phylogenetic analysis based on near full-length 16S rRNA gene sequences showed that the four PGPB strains for duckweed belonged to the phylum *Proteobacteria* (note that a renaming of this phylum to *Pseudomonadota* has recently been proposed) (Table 1). The sequences of two strains, MRB1 and MRB3, showed high identity (99.2–99.3%) to that of *Pelomonas saccharophila* DSM654^T^ (previously described as *Pseudomonas saccharophila* [57]) in the class *Betaproteobacteria*. Although the sequences of these two strains were completely identical, their PGP traits differed at the strain level, as discussed later. MRB4 also shared a high sequence identity (99.6%) with *Bradyrhizobium guangdongense* CCBAU51649^T^ (the class *Alphaproteobacteria*). In contrast to the other strains, the 16S rRNA gene sequence of MRB2 belonging to the family *Rhodanobacteraceae* of the class *Gammaproteobacteria* exhibited low identity (<94%) with the sequences of the closest type strains of the known genera *Aquimonas*, *Fulvimonas*, and *Dokdonella*. This suggests that MRB2 is phylogenetically novel, at least at the genus level.

The betaproteobacterial *Pelomonas* species, which are closely related to strains MRB1 and MRB3, were detected in the rhizoplane of *Lemna gibba* [58] as well as the roots of common reed (*P. australis*) [27]. Intriguingly, they were also detected in the rhizosphere or endosphere of various terrestrial plants, such as sorghum [59], sweet potato [60], and rice [61]. On the other hand, *Pelomonas* spp. have not been reported as PGPB for terrestrial or aquatic plants; to the best of our knowledge, therefore, our *Pelomonas* strains isolated here are the first representatives identified as PGPB. Among the *Pelomonas* species, the type strain of *P. saccharophila*, which was one of the nearest strains of MRB1 and MRB3, possesses both hydrogen-oxidizing and nitrogen-fixing abilities [57]. Several aquatic *Pelomonas* species have been reported to lack both abilities (e.g., *Pelomonas aquatica* CCUG 52575^T^ [62]), but strains MRB1 and MRB3 had a nitrogen fixation capability, as described below.

The alphaproteobacterial *Bradyrhizobium* spp., which are closely related to strain MRB4, are well documented to promote the growth of terrestrial leguminous and non-leguminous plants [63,64]. In this context, our *Bradyrhizoubium* sp. MRB4 is a rare example of a bacterium with a growth-promoting effect on aquatic plants, and no root nodules and nodule-like structures were observed in the *Lemna*/MRB4 co-culture (data not shown). It is worth noting that other members in the order *Rhizobiales* (for which the new name *Hyphomicrobiales* was recently proposed), including the genera *Devosia*, *Ensifer*, *Mesorhizobium*, *Methylobacterium*, and *Rhizobium*, have been recovered from various duckweeds, such as *L. aequinoctialis* [65,66], *L. minor* [22], *Lemna japonica* [67], and *S. polyrhiza* [26], as well as other aquatic plants [27]. However, none of these members are known as aquatic PGPB, with the exception of *Allorhizobium* species, which promote nodulation in the aquatic leguminous plant *Neptunia natans* [68]. Further exploration of the association of *Rhizobiales* members with aquatic plants may lead to the discovery of novel symbiotic combinations.

**Figure 3:**
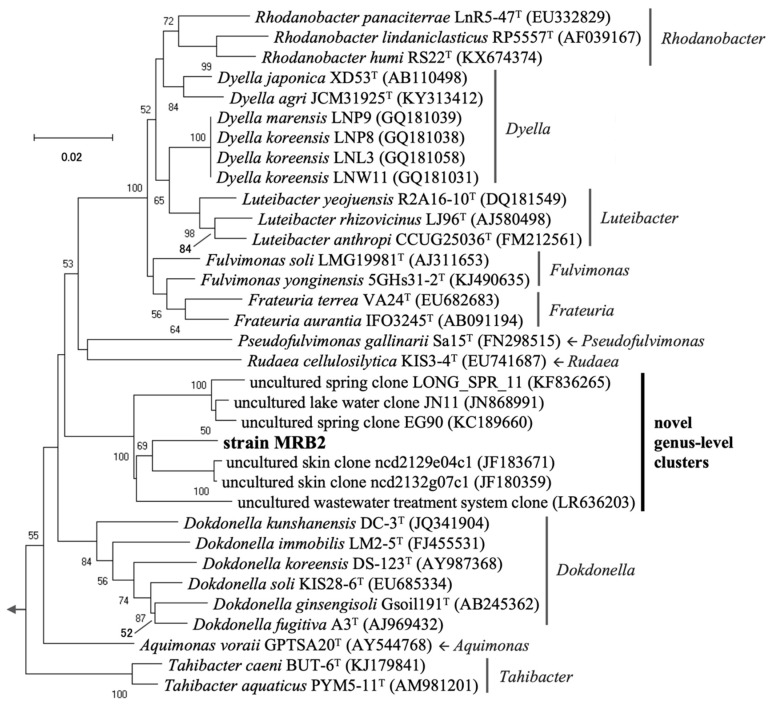
Phylogenetic tree based on near full-length 16S rRNA gene sequences from a novel strain MRB2 and its related type species, known plant growth-promoting bacteria (PGPB), and environmental sequences. The evolutionary relationship in the family *Rhodanobacteraceae* (class *Gammaproteobacteria)* was inferred using the neighbor-joining method with the Kimura 2-parameter model. There were a total of 1257 positions in the final dataset. *Pseudomonas aeruginosa* ATCC10145^T^ was used as an outgroup. Accession numbers of nucleotide sequences registered in the DDBJ/ENA/NCBI databases are shown in parentheses. Bootstrap values >50% based on 1000 replicates are shown at the nodes. Scale bar, 0.02 nucleotide substitutions per site.

The novel strain MRB2 showed relatively low sequence identity (<97%) even to the uncultured environmental clones retrieved from human skin (accession numbers JF180359 and JF183671), spring water (KC189660 and KF836265), and lake water (JN868991). In agreement with this result, strain MRB2 formed an independent cluster on the phylogenetic tree of the family *Rhodanobacteraceae* (Figure 3). Focusing on this family, members belonging to several genera have been reported as terrestrial PGPB; *Rhodanobacter* sp. MTR-45B as an efficient PGPB for quailbush and buffalo grass [69], *Dyella* spp. for *Lespedeza* sp. [70], *Luteibacter rhizovicinus* MIMR1 for barley [71], *Frateuria aurantia* for tobacco [72], and *Dokdonella* spp. for wheat [73]. These data indicate that this family tends to contain multiple PGPB strains, including our novel strain MRB2, for a variety of plants.

### 3.3. Characterization of Plant Growth-Promoting Properties

We tested four PGP activities—i.e., IAA production, siderophore production, phosphate solubilization, and nitrogen fixation—all of which have been well observed in terrestrial PGPB [29]. All our strains MRB1–4 showed a capacity for IAA production when l-tryptophan, the precursor of IAA, was added; most of the other PGPB are also known to produce IAA (Table 2). Note that l-tryptophan is one of the main exudates of various plants (e.g., tomato, cucumber, and radish [74]), and tryptophan-like compounds are also thought to be present in *L. minor* exudates [20]. IAA production is the major PGP factor of terrestrial PGPB, while its effectiveness on aquatic plants is still uncertain, as noted above (i.e., one study found that exogeneous IAA had no positive effect on *L. minor* growth [28]). However, since most of the endophytic bacteria isolated from duckweeds were reported to be present in an IAA production assay [75], it may be that internal IAA production is important for the growth promotion for duckweeds.

The traits of siderophore production and phosphate solubilization varied among strains MRB1–4; this trend has also been observed in other PGPB for duckweeds (Table 2). Siderophore production activity was found in three strains, MRB-2, -3, and -4. *Pelomonas* strains MRB1 and MRB3 shared an identical 16S rRNA gene sequence, while only MRB3 was positive in the production assay. Siderophores produced by PGPB have attracted attention for their ability to enhance the solubility and plant-availability of oxidized ferric iron in the soil environments [76,77]. Similarly, since inorganic phosphorus is present in insoluble forms (e.g., di- and tri-calcium phosphates) [78], the phosphate solubilization by PGPB is also important in soils. All of our strains lacked solubilization ability in our solubilization assays, while Ishizawa et al. [22] demonstrated using the same medium as used in this study (mHoagland solution) that duckweed growth promotion slightly correlated only with the phosphate-solubilizing ability of bacterial isolates. Compared with soil environments, both iron and inorganic phosphorus are considered to be well solubilized in water, and their solubilization abilities by PGPB may not be crucial factors for the aquatic host growth promotion.

We further found that *Pelomonas* strains MRB1 and MRB3 possessed a key gene for nitrogen fixation (*nifH*) (Table 2). As mentioned above, the known *P. saccharophila* had a capacity to fix nitrogen gas [48]. The *nifH* gene sequences of MRB1 and MRB3 exhibited high amino acid identity (96.7–96.8%) with that of *P. saccharophila* (accession no. BAE15986). Strains MRB1 and MRB3 also showed growth in nitrogen-free medium. Note that the sequence of *Bradyrhizobium* strain MRB4 also showed a high identity (99.2%) to the *nifH*-like gene sequence of *Bradyrhizobium betae* (accession no. WP_151648738). Importantly, nitrogen fixation by *Pelomonas* and *Bradyrhizobium* species is reported to be stimulated under microaerobic conditions [48,79]. Moreover, *Bradyrhizobium* spp. form specialized root nodules for nitrogen fixation [80,81], but no such nodule structures were observed in this study. Given that duckweeds perform photosynthesis under light and provide aerobic conditions, it is logical to assume that our PGPB strains do not exhibit high nitrogen-fixing activity during co-culture. On the other hand, because one of our strains, strain MRB3, can form local biofilms on the host *L. minor* surfaces (see below), it may be that the biofilms create an oxygen-depleted microenvironment facilitating nitrogen fixation activity. Future studies will be needed to verify this point.

**Table 2:** Plant growth-promoting factors and motility of the obtained strains MRB1–4 and known PGPB for duckweeds. IAA: indole-3-acetic acid. The symbols indicate the following: +, positive; +/−, slightly positive; −, negative; nd, no data.

Taxon (Phylum or Class)	Aquatic PGPB Strains	IAA	Siderophore	Phosphate Solubilization	Nitrogen Fixation	Motility	Reference
*Acidobacteria*	*Paludibaculum* sp. F-183	+	−	−	nd	nd	[24,82,83]
	*Luteitalea* sp. TBR-22	−	−	−	nd	nd	
*Alphaproteobacteria*	*Bradyrhizobium* sp. MRB4	+	+	−	− *	+	this study
	*Ensifer* sp. SP4	−	+	−	−	nd	[23]
*Betaproteobacteria*	*Pelomonas* sp. MRB1	+	−	−	+	+	this study
	*Pelomonas* sp. MRB3	+	+	−	+	+	this study
	*Aquitalea magnusonii* H3	+	+	+	nd	+	[22,84]
*Gammaproteobacteria*	*Rhodanobacteraceae* sp. MRB2	+	+	−	−	−	this study
	*Acinetobacter calcoaceticus* P23	−	+	+	nd	+ ^†^	[18,85]
	*Pseudomonas* sp. Ps6	+	−	+/−	nd	+ ^†^	[18]
	*Pseudomonas otitidis* M12	+	+	+	nd	nd	[22]
*Firmicutes*	*Bacillus amyloliquefaciens* FZB42	+	+	+	nd	+	[20,86,87,88]
*Bacteroidetes*	*Chryseobacterium* sp. 27AL	+	+	−	−	nd	[85]
	*Chryseobacterium* sp. 29AL	+	+	−	−	nd	

* A *nifH*-like gene sequence was detected by PCR in this study. ^†^ The motility of these bacteria was assessed as swarming motility on solid medium.

### 3.4. PGPB Adhesion and Colonization of Duckweed Surfaces

**Figure 4:**
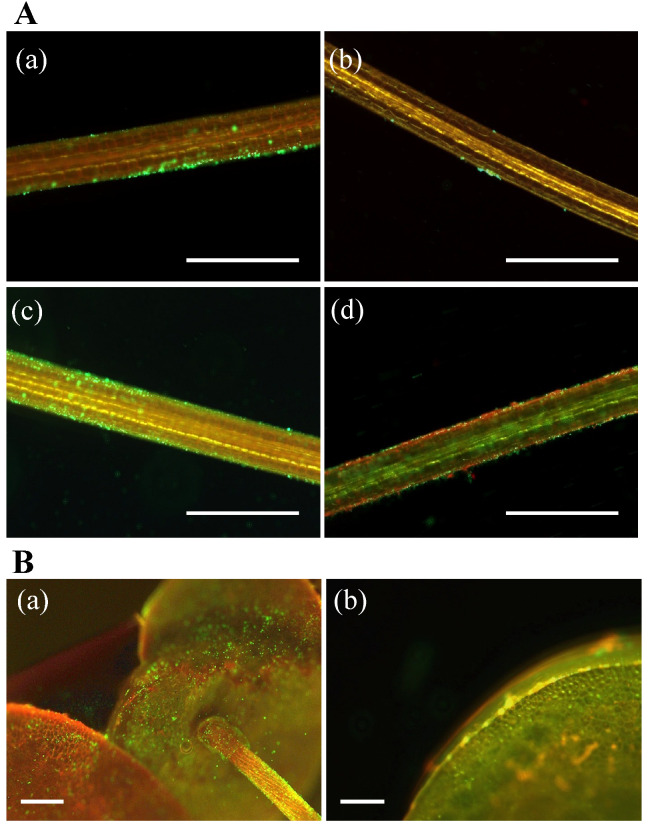
Fluorescent micrographs of LIVE/DEAD-stained bacterial cells attached on roots and fronds of *Lemna minor* co-cultured with the PGPB strains MRB1–4. (**A**): Micrographs show the duckweed roots harboring strains (**a**) MRB1 at day 14, (**b**) MRB2 at day 3, (**c**) MRB3 at day 14, and (**d**) MRB4 at day 3. Scale bars, 200 µm. (**B**): Micrographs exhibit the duckweed harboring MRB3 (**a**) on the frond ventral side of the root at day 3 and (**b**) on the frond dorsal side at day 14. Scale bars, 250 µm. All cells shown here were stained green using SYTO9 for viable cells or red using propidium iodide for dead cells.

Adhesion to plant surfaces is considered one of the key activities by which PGPB sustain a plant–microbe interaction [5,18,19,89,90]. During the initial adhesion process, the motility of bacteria is important for their adhesion to the host surface. Through microscopic observations, three of the PGPB strains examined here, but not MRB2, were found to possess cell motility (Table 2). We further found that viable cells were present in the *Lemna*/PGPB co-cultures on days 3–14, based on positive staining with the LIVE/DEAD BacLight Bacterial Viability Kit (Figure 4). As discussed below, cells of strains MRB1, -3, and -4 were attached to the host *Lemna* surfaces (Figure 4A(a,c,d)), while most of the cells of strain MRB2 did not attach to the host (Figure 4Ab), which is consistent with their lack of motility. It should be noted that strain MRB2 exerted the greatest effect on increasing the biomass and chlorophyll content of duckweed (Figure 1c,d). This strain perhaps may have a PGP (e.g., diffusible factor) that is not present in the other three strains, and that is not mediated by a strong adhesion.

Many cells of *Pelomonas* strain MRB3, which showed the highest growth-promoting effect for duckweed in this study (Figure 1a), were able to colonize the host *L. minor* root surfaces and form local biofilms (Figure 4Ac). We also found that MRB3 cells adhered to the root on all observation days (days 1, 3, 7, 10, and 14; Figure S1). The *Pelomonas* strain MRB1 also attached to the duckweed root (Figure 4Aa). Adhesion properties were previously observed with the known PGPB strains for duckweed, *A. calcoaceticus* P23 [17], *B. amyloliquefaciens* FZB42 [91], *Pseudomonas* sp. Ps6 [18], *A. magnusonii* H3 [22], *Paludibaculum* sp. F-183 [24,82], and *Luteitalea* sp.TBR-22 [24,83], during co-culture periods ranging from 3 to 10 days. Physical attachment is thus likely to be the key characteristic of aquatic PGPB and might be more important in aquatic environments with constant water flow. Further observation showed that strain MRB3 colonized not only the root surfaces but also the frond ventral and dorsal sides (Figure 4B(a,b)). Such an extensive colonization was also observed from the association between *B. amyloliquefaciens* FZB42 and duckweed [91]. In contrast to strains MRB1 and MRB3, *Bradyrhizobium* strain MRB4 also showed an adhesion capability, but more than half of the MRB4 cells were dead, as evidenced by their red fluorescence in LIVE/DEAD staining (Figure 4Ad). Given that this strain showed relatively low growth promotion in this study (Figure 1), the survivability of the attached cells might be related to the performance of the growth-promoting effect.

**Figure 5:**
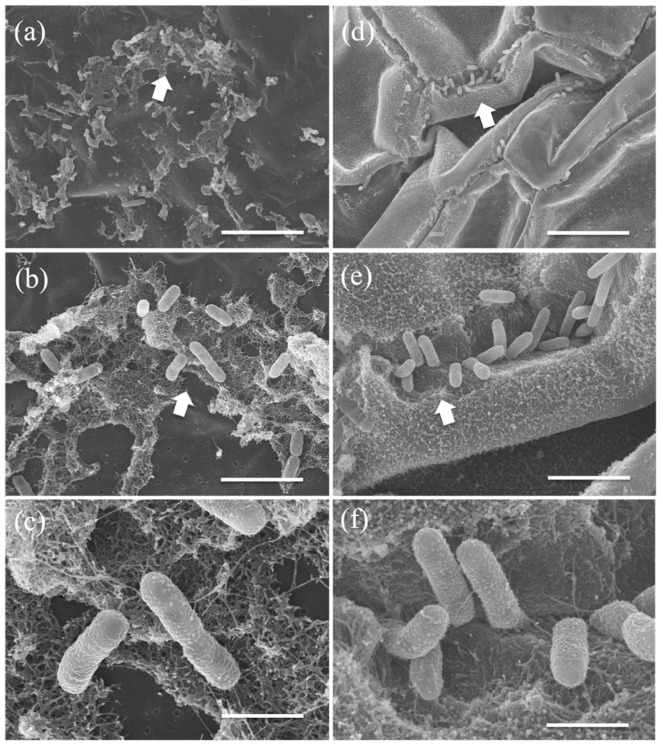
Scanning electron micrographs of bacterial cells attached on the frond and root surface of *Lemna minor* co-cultured with MRB3. Cells (rod shape) of strain MRB3 adhered to and colonized (close-ups of arrows from (**a**–**c**)) the frond ventral side and (close-ups from (**d**–**f**)) the root surface at day 7. Scale bars: (**a**) and (**d**), 10 μm; (**b**) and (**e**), 3 μm; (**c**) and (**f**), 1 μm.

Scanning electron microscopic observation of the *Lemna*/MRB3 co-culture revealed that strain MRB3 formed local biofilms on the *L. minor* frond (Figure 5a,b) and produced fiber-like extracellular substances (Figure 5c). A similar fiber-like structure was also observed around the cells embedded on the root surface, although this structure was not well developed (Figure 5d–f). Extracellular secretions are important for bacterial adhesion [92]; moreover, secreted substances (e.g., extracellular polysaccharides) are considered to protect host plants against environmental stressors such as desiccation [93]. It is unclear whether *Pelomonas* spp. adhere to aquatic plants in natural environments, but the circumstantial evidence includes the following reports: *Pelomonas* spp. were detected on natural duckweed [58] and common reed (*P. australis*) [31] by culture and molecular methods; the 16S rRNA gene sequences of our *Pelomonas* strains MRB1 and MRB3 showed a high sequence identity (>99%) with those of isolates (accession nos. LC378793, LC378785, and LC378788) enriched when the aseptic duckweed *S. polyrhiza* was co-cultured with aquatic microorganisms [94]. Taken together with these previous reports, our present results suggest that *Pelomonas* members may be associated with duckweed plants in nature.

## 4. Conclusions

In the present study, we isolated and characterized four novel aquatic PGPB strains, including the previously overlooked *Pelomonas* strains and a novel genus-level strain. These strains increased the growth, biomass, and chlorophyll content of their host, duckweed. Moreover, the PGP traits in our strains included IAA production, which is also observed in known terrestrial and aquatic PGPB strains, while other traits, such as siderophore production and phosphate solubilization, varied among the strains. Moreover, *Pelomonas* strains displayed a strong capacity for adhesion to the frond and root surface of the duckweed. Because the promotion factors and traits required for PGPB differ between soil and aquatic environments, further study of the duckweed PGPB obtained herein could lead to the elucidation of unforeseen mechanisms underlying aquatic plant-microbe interactions.

## 5. Patents

The four strains obtained in this study were deposited in the National Institute of Technology and Evaluation (NITE, Chiba, Japan), NITE Patent Microorganisms Depository (accession numbers: NITE P-01645–P-01648).

## Figures and Tables

**Table 1:** Phylogenetic affiliation of 16S rRNA gene sequences of the novel PGPB strains MRB1–4, with the top three BLASTN hits against the EzBioCloud database.

Strain	Taxon	Closest Type Strain (Accession Number)	Isolation Source	Identity (%)
MRB1	*Betaproteobacteria*	*Pelomonas saccharophila* DSM654^T^ (SMBU01000080)	mud	99.22
		*Pelomonas aquatica* CCUG52575^T^ (AM501435)	industrial water	99.21
		*Pelomonas puraquae* CCUG52769^T^ (NISI01000035)	hemodialysis water	99.15
MRB2	*Gammaproteobacteria*	*Aquimonas voraii* DSM16957^T^ (jgi.1058856)	warm spring water	93.82
		*Fulvimonas soli* LMG19981^T^ (AJ311653)	soil	93.58
		*Dokdonella soli* KIS28-6^T^ (EU685334)	mud	93.51
MRB3	*Betaproteobacteria*	*Pelomonas saccharophila* DSM654^T^ (SMBU01000080)	mud	99.27
		*Pelomonas aquatica* CCUG52575^T^ (AM501435)	industrial water	99.25
		*Pelomonas puraquae* CCUG52769^T^ (NISI01000035)	hemodialysis water	99.12
MRB4	*Alphaproteobacteria*	*Bradyrhizobium guangdongense* CCBAU51649^T^ (KC508867)	nodules of *Dalbergia*	99.58
		*Bradyrhizobium centrosematis* A9^T^ (KC247115)	nodules of *Centrosema*	99.53
		*Bradyrhizobium ganzhouense* RITF806^T^ (JQ796661)	nodules of *Acacia*	99.53

## Data Availability

The sequence data obtained in this study have been deposited in the DDBJ/ENA/NCBI databases under accession numbers LC710946 to LC710949.

## Supplementary Materials

The following supporting information can be downloaded at https://www.mdpi.com/article/10.3390/microorganisms10081564/s1: Figure S1, Fluorescent micrographs of LIVE/DEAD stained MRB3 cells attached to duckweed *Lemna minor* during co-culture.
