# Supplementary material for: Isolation of Aquatic Plant Growth-Promoting Bacteria for the Floating Plant Duckweed (Lemna minor)
Source: Microorganisms. 2022 Aug 3;10(8):1564. doi: 10.3390/microorganisms10081564 (PMC9416352; doi:10.3390/microorganisms10081564)
Supplement: Supplementary file 1 [file microorganisms-10-01564-s001.zip › microorganisms-1813772-supplementary.pdf]

Supplementary Figure S1:

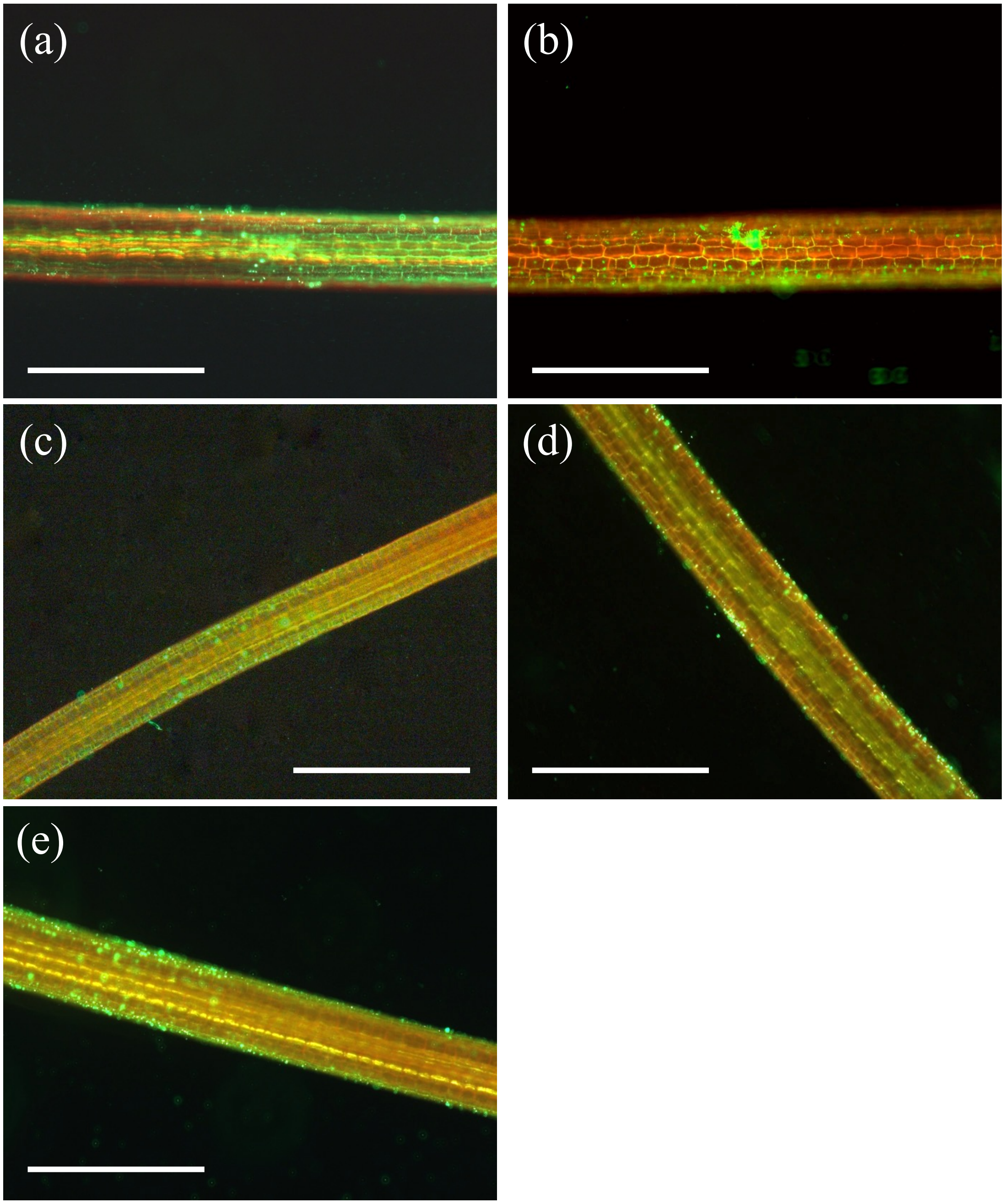

Figure S1. Fluorescent micrographs of LIVE/DEAD-stained MRB3 cells attached to duckweed *Lemna minor* during co-culture. Micrographs show the duckweed roots harboring MRB3 at day (a) 1, (b) 3, (c) 7 (d) 10 and (e) 14. The cells were distinguished in green using SYTO 9 for viable cells or in red using propidium iodide for dead cells. Scale bars, 200 μm.
